# The biosecurity busyness paradox: Why doing more and remaining consistent may be undermining global resilience

**DOI:** 10.1016/j.onehlt.2026.101558

**Published:** 2026-09-06

**Authors:** Justin I. McDonald

**Affiliations:** Aquatic Biosecurity, WA Department of Primary industries and Regional Development (DPIRD), 39 Northside Drive, Hillarys 6025, Western Australia, Australia

Globally, we are all part of a rapidly changing, interconnected, and interdependent world. Increased connectivity, climate change, food and water security issues, global pandemics, and habitat decline, are all contributing to an evolving and increasingly stressful risk landscape. As human activity reshapes ecosystems, redistributes species and intensifies interactions between human, animal and environmental health, biosecurity has become one of the defining challenges of the Anthropocene and a foundational component of planetary one health.

There are numerous examples of increasing biosecurity pressures, from the now global coverage of Highly Pathogenic Avian Influenza [1] and its crossover into dairy cattle in the United States [2], to the expansion of the Dengue vector in Europe [3], to the far-reaching impacts of Polyphagous shot hole borer in Western Australia [4] and the constant threat of foot and mouth disease [5].

Governments the world over are striving to adapt and translate limited public-sector resources into better outcomes. However, many often fail in their application of change [6]. Long shaped by assumptions of containment, control and rapid response, these systems are increasingly strained by the convergence of ecological disruption, accelerating global connectivity and finite institutional capacity. What is emerging is not simply a rise in biosecurity risk, but a systemic vulnerability: a growing mismatch between the scale of global threats and the way biosecurity systems are historically designed to respond.

The issue is not that governments are working too little. Quite the opposite. Biosecurity professionals are responding to more incursions than ever before. Current staff resource limitations, rising workloads, and increasingly complex threats already threaten current systems. Simply adding more resources to existing models is unlikely to provide a solution. At Australian borders alone the annual number of interceptions of biosecurity risk materials rose by almost 50% between 2012 and 2017. The concern is that systems have become so occupied by responding that they have little opportunity to ask whether they are responding to the right problems in the right way. CSIRO [7] state “continuing along the ‘business as usual’ trajectory of slow and incremental change could expose Australia to significant triple bottom line risks over the next 10 years”. Scaling the current system through additional funding allocation will not be enough. Modelling shows that even almost tripling investment in interventions out to 2025 will still result in increased residual biosecurity risk. While increased resourcing is essential to ensure we ‘keep up’ we drastically need to rethink our systems and how they operate.

At the core of this challenge lies what can be termed the biosecurity busyness paradox: the more biosecurity systems do, the less capable they may become of addressing the underlying drivers of risk. The issue is not that we are doing too much, but rather that we are not pausing and thinking about what we do, and why. Under increasing workload and constrained resources, this leads to a self-reinforcing cycle. Systems become optimised for responsiveness, while the capacity for critical thinking, prioritisation and long-term planning is progressively eroded. This pattern reflects a broader human tendency in modern economies, where busyness is mistaken for productivity and where there is an erosion of time dedicated to strategic, long-term, and critical thinking [8], [9].

High operational tempo, constant task engagement and limited workforce can induce cognitive overload, narrowing attention to immediate demands, and diminish capacity for strategic thinking and problem solving. A process often described as cognitive tunnelling [10]. In the context of biosecurity, this tendency manifests as a relentless cycle of reactive responses - jumping from one crisis to another without the space for strategic evaluation. Operational surveillance, interception, and eradication remain essential. However, an overemphasis on immediate response has created a structural and cultural imbalance in which action is prioritised over reflection, throughput over strategy, and compliance over adaptation. In such environments, decision-makers are less able to evaluate trade-offs, challenge assumptions or identify emerging risks over the long term. Workers “go on automatic,” following established rules and procedures without questioning their effectiveness [11]. Highly reactive organisations become trapped in a cycle of managing today's crisis while unintentionally increasing their vulnerability to tomorrow's. This matters because the drivers of biosecurity risk are changing faster than the institutions designed to manage them.

Equally important is creating institutional space for thought. Bureaucratic systems, particularly those managing risk, are often structured to reward compliance, throughput, and adherence to established procedures rather than creativity or anticipatory thinking [12], [13]. Consequently, organisations often become highly efficient at repeating existing practices, whether appropriate or not, even as the operating environment changes around them. By embedding reflection, prioritisation and foresight into the core of biosecurity governance, it is possible to move from a reactive to a proactive paradigm, one that not only responds to threats but anticipates and mitigates them. Reflective practice must be embedded within governance structures, not treated as an optional add-on. This includes creating structured space for strategic dialogue, integrating systems thinking into training and decision-making, and developing performance frameworks that reward learning, innovation and long-term impact. Horizon scanning, scenario planning, systems modelling and post response evaluation should become core operational functions rather than occasional exercises. Pausing to think becomes the foundation of better action.

We cannot continue to do everything we have done with the same structures, the same risk appetites and with the same resourcing. We need to change the operating model and rethink how we view and respond to risk. We also need governments to acknowledge increasing risk and dig deeper into their treasuries and consider how they prioritise public sovereignty and long-term security. Not all biosecurity threats carry equal risk, yet many systems continue to allocate resources in ways that implicitly assume they do. Biosecurity must also be reconceptualised as a shared societal responsibility. Governments alone cannot manage the scale and complexity of contemporary biosecurity risk. Industry actors, who are often both contributors to and beneficiaries of biosecurity systems, have a critical role to play in managing sector-specific risks. A more strategic approach would explicitly differentiate between threats to the biosecurity “common good”, those affecting environmental integrity, public health and shared societal values, and those that are more appropriately managed at the sectoral or industry level. Such differentiation enables more efficient allocation of limited public resources while maintaining overall system effectiveness.

The greatest threat facing biosecurity may therefore not be the next invasive species or emerging disease. It may be our continued reliance on governance models designed for a simpler world. A One Health approach provides an opportunity to rethink how biosecurity is governed [14]. Although One Health has traditionally focused on the interconnectedness of human, animal and environmental health, its principles are equally applicable to biosecurity. Healthy ecosystems underpin food security, biodiversity, clean water, economic prosperity and human wellbeing. Most emerging biosecurity threats develop across these domains simultaneously. One Health encourages systems thinking. Environmental surveillance becomes an early warning system for both ecological and human health threats. Wildlife monitoring informs disease preparedness. Ecosystem health becomes a predictor of future biosecurity risk rather than simply an environmental outcome.

One Health should no longer be regarded as an aspirational concept. In an increasingly interconnected world, it is a practical necessity for building resilient biosecurity systems capable of protecting both people and the planet. Biosecurity is not an isolated technical function; it is integral to maintaining the stability of ecological systems, safeguarding food security and preventing the spread of emerging diseases. As such, the limitations of current biosecurity models have implications that extend beyond national borders, intersecting with global sustainability transitions.

However, the pathways forward are clear. The enabling factors are targeted and integrated science investment, refined surveillance, better data integration and use, better systems understanding that translates into operations. Ensure adequate human resourcing for long-term consistent capacity and capability and investigate alternate risk appetites. Finally, and perhaps most importantly redesign business structures to create the space to think, in systems that have become conditioned only to do.

## CRediT authorship contribution statement

**Justin I. McDonald:** Writing – review & editing, Writing – original draft, Conceptualization.

## Funding

The author has nothing to report.

The author declares no conflicts of interest.

## Declaration of competing interest

The authors declare that they have no known competing financial interests or personal relationships that could have appeared to influence the work reported in this paper.

## Data Availability

Data sharing not applicable to this article as no datasets were generated or analysed during the current study
